# Immunological and Metabolomic Impacts of Administration of Cry1Ab Protein and MON 810 Maize in Mouse

**DOI:** 10.1371/journal.pone.0016346

**Published:** 2011-01-27

**Authors:** Karine Adel-Patient, Valeria D. Guimaraes, Alain Paris, Marie-Françoise Drumare, Sandrine Ah-Leung, Patricia Lamourette, Marie-Claire Nevers, Cécile Canlet, Jérôme Molina, Hervé Bernard, Christophe Créminon, Jean-Michel Wal

**Affiliations:** 1 INRA, UR496, Unité d'Immuno-Allergie Alimentaire, Jouy-en-Josas, France; 2 INRA, UR1204, Méthodologies d'Analyse de Risque Alimentaire, Paris, France; 3 CEA, iBiTeC-S, Laboratoire d'Etude et de Recherche en Immunoanalyse, Gif sur Yvette, France; 4 INRA, UMR1089, Xénobiotiques, Toulouse, France; National Institute of Environmental Health Sciences, United States of America

## Abstract

We have investigated the immunological and metabolomic impacts of Cry1Ab administration to mice, either as a purified protein or as the Cry1Ab-expressing genetically modified (GM) MON810 maize. Humoral and cellular specific immune responses induced in BALB/cJ mice after intra-gastric (i.g.) or intra-peritoneal (i.p.) administration of purified Cry1Ab were analyzed and compared with those induced by proteins of various immunogenic and allergic potencies. Possible unintended effects of the genetic modification on the pattern of expression of maize natural allergens were studied using IgE-immunoblot and sera from maize-allergic patients. Mice were experimentally sensitized (i.g. or i.p. route) with protein extracts from GM or non-GM maize, and then anti-maize proteins and anti-Cry1Ab–induced immune responses were analyzed. In parallel, longitudinal metabolomic studies were performed on the urine of mice treated via the i.g. route. Weak immune responses were observed after i.g. administration of the different proteins. Using the i.p. route, a clear Th2 response was observed with the known allergenic proteins, whereas a mixed Th1/Th2 immune response was observed with immunogenic protein not known to be allergenic and with Cry1Ab. This then reflects protein immunogenicity in the BALB/c Th2-biased mouse strain rather than allergenicity. No difference in natural maize allergen profiles was evidenced between MON810 and its non-GM comparator. Immune responses against maize proteins were quantitatively equivalent in mice treated with MON810 *vs* the non-GM counterpart and no anti-Cry1Ab–specific immune response was detected in mice that received MON810. Metabolomic studies showed a slight “cultivar” effect, which represented less than 1% of the initial metabolic information. Our results confirm the immunogenicity of purified Cry1Ab without evidence of allergenic potential. Immunological and metabolomic studies revealed slight differences in mouse metabolic profiles after i.g. administration of MON810 *vs* its non-GM counterpart, but no significant unintended effect of the genetic modification on immune responses was seen.

## Introduction

Food allergies, mainly IgE-mediated immediate reactions, are increasing worldwide, particularly in Western countries. The most common food allergens include peanut, soybean, tree nuts, wheat, egg, milk, fish and sea foods, but many other foods may be involved [1], [2] and the prevalence of allergy to particular foods varies in different geographic areas owing to dietary habits and environmental conditions. The introduction on the market of novel foods, particularly foods resulting from modern biotechnology, e.g. genetically modified (GM) foods, has therefore raised the question of the assessment of the potential allergenicity of the newly expressed protein(s) and of the whole GM food. As no single test or property definitely distinguishes allergens from non-allergens, the allergenicity of a novel protein is currently assessed using a weight-of-evidence approach [3] [4] [5] [6]. Although called into question [7], the use of animal models has been encouraged by international scientific committees to complement this approach.

Various animal models have been proposed for allergenicity assessment (review in [8]). Mice have been widely used because they share with humans many important immunological mechanisms, such as Th1, Th2, Th17 and regulatory responses [9] [10]. Many immunological studies have been performed with BALB/c mice, a Th2-biased high IgE responder strain mimicking atopic individuals [11]. BALB/c mice have been used for the study of both steps of the allergic reaction to various food allergens, i.e. sensitization (the synthesis of specific IgE antibodies) and elicitation (the appearance of symptoms upon challenge of sensitized animals) [12], [13] [14], [15]. It has been proposed that the intrinsic sensitizing potential of a novel protein can be assessed by measuring the specific IgE antibody and Th2 cytokine productions after administration without adjuvant. However, BALB/c, like other inbred congenic mice, are characterized by a defined and restricted haplotype and false-negative IgE production can be observed due to non-recognition of the administered proteins by the class II major histocompatibility complex. The capacity of a protein to induce the synthesis of IgG antibodies, such as IgG1 or IgG2a antibodies which are Th2 or Th1 markers respectively, should also be measured for a comprehensive assessment [8]. Additionally, the comparison between the immune response induced by administration of the novel protein and that induced by a range of different proteins known to be weak or strong sensitizers has been proposed to increase the sensitivity and specificity of the test and the accuracy of the interpretation [16], [17].

*Bacillus thuringiensis* (*Bt*), a gram-positive bacterium producing insecticidal inclusion bodies during sporulation, has been widely used in insecticidal spray and some of its genes have been introduced into many crops to make them resistant to insect infestations. Among the different *Bt* proteins, Cry1Ab has been introduced by genetic modification in various crops including so-called insect-resistant maizes such as MON810. Because of the specificity of the insect gut receptors, *Bt* proteins are considered innocuous to mammals [18]. Cry1Ac, which has a structure very close to that of Cry1Ab, has been shown to be immunogenic in BALB/c mice, inducing a systemic and a mucosal immune response with production of specific IgG, IgM and IgA antibodies after i.p., i.g., intra-nasal or intra-rectal administration [19], [20]. The N-terminal region of Cry1Ab, Cry1Ac and also of Cry1Aa, another similar protein, may induce strong specific antibody responses after intra-nasal or i.p. administration to BALB/c mice, and Cry1Aa was shown to induce cytokine production in mouse lymphocytes [21]. The induction of a specific immune response was also observed in rats after ingestion of transgenic rice containing or spiked with Cry1Ab protein [22] and 2 human T-cell epitopes of Cry1Ab have been identified *in vitro* [23]. In addition, a recent study suggested that feeding weaning and old mice with MON810 maize may result in alterations of intestinal and peripheral immune cell populations [24]. All these studies demonstrate the intrinsic immunogenicity of Cry1A proteins, although specific IgE were not assessed in animals experimentally treated and never detected in sera from humans with various allergies who might have been exposed to insect-resistant crops [25]–[27].

We assessed the intrinsic immunogenic/allergenic potential of Cry1Ab in BALB/c mice after administration of the purified protein by different routes without adjuvant. We compared the specific antibody responses and cytokine secretions thus induced with those induced after administration of other proteins known to display different immunogenic/allergic potencies [14], [16], i.e. keyhole limpet hemocyanin (KLH), considered as an immunogen but not known to be an allergen, cow's milk bovine β-lactoglobulin (BLG), which is a moderate allergen, and peanut Ara h 1, which is known as a most potent allergen. Cry1Ab is present at very low levels in maize seeds and so we also analyzed the immune response induced in mice after administration of protein extracts from maize MON810 and its non-GM counterpart. Both maize protein extracts were first characterized for their allergen contents and profiles and Cry1Ab contents and then administered to mice via the i.p. or i.g. route. Additionally, longitudinal metabolomic studies were also performed in the mice treated (i.g. route) with GM *vs* non-GM maize. Metabolomics complements the immunological studies. As a non-targeted study, we used it to detect any unintended effects of the genetic modification on the metabolism of mice given MON810 *vs* non-GM maize protein extract. Such metabolic changes may be explained by minor perturbations or deregulations in the metabolic network in response to exposure to these protein extracts *via* the i.g. route.

## Methods

### 1. Apparatus, reagents, mice and ethics statement

Enzyme immunometric assays were performed in 96-well microtiter plates (Immunoplate Maxisorb, Nunc, Roskilde, Denmark) using specialized Titertek microtitration equipment from Labsystems (Helsinki, Finland). Unless otherwise stated, all reagents were of analytical grade from Sigma (St Louis, MO).

Female BALB/cJ mice (Centre d'Elevage René Janvier, France) were housed under normal husbandry conditions and were acclimated for two to three weeks before experimentation. All experiments were performed according to the European Community rules of animal care and with permission 91–122 of the French Veterinary Services. All experiments were covered by agreement No. 2009-DDSV-074 (October 29th, 2009) from the Veterinary Inspection Department of Essonne (France).

### 2. Production and characterization of anti-Cry1Ab monoclonal antibodies

Anti-Cry1Ab monoclonal antibodies (mAbs) were produced by conventional techniques according to De StGroth and Scheidegger [28] and Grassi et al. [29] using Cry1Ab full-length protein purified from *Bacillus thuringiensis* (i.e. protoxin, see section 2.3.1). Twenty-five mAbs were obtained 20 of which were of IgG1 isotype and 4 of IgG2a isotype. Five complementarity groups, i.e. recognizing different regions of Cry1Ab protein, were characterized, one of which was specific to the C-terminal part of the protoxin [30]. Two complementary mAbs (i.e. mAb#120 and mAb#95) were selected based on their epitopic specificity and affinity, for ELISA determination of Cry1Ab. In addition, mAbs from different complementarity groups were used as standards for the quantification of anti-Cry1Ab IgG1 and IgG2a antibodies produced in mice after i.p. or i.g. administration (see below).

### 3. Evaluation of the intrinsic immunogenicity of purified Cry1Ab

#### 3.1 Purified proteins

Extraction and purification of bovine β-lactoglobulin (BLG) from cow's milk were performed as previously described [31]. Ara h 1 was extracted and purified from roasted peanuts according to [32]. Cry1Ab protoxin was produced from the *B. thuringiensis* 407- strain harboring the pHT315Ωcry1Ab plasmid [30], [33]. Identity, purity and molecular weight (MW) of all proteins were confirmed by HPLC using Akta purifier and SDS-PAGE electrophoresis systems from Pharmacia Biotech, completed by in-gel trypsin hydrolysis and analysis of generated fragments by MALDI-TOF mass spectrometry (data not shown; [30]). KLH was from Calbiochem (La Jolla, CA). Protein concentrations were assessed using BCA assay from Pierce. No endotoxin was detected in protein preparations, as determined by the Limulus Amebocyte Lysate test from Sigma.

#### 3.2 Analysis of the immune response induced after administration of purified proteins

Seven-week-old female BALB/cJ mice were given either 1 or 100 µg of each protein (i.e. KLH, BLG, Ara h 1 or Cry1Ab) by the i.p. or i.g. route at days 1 and 15 without any adjuvant (*n* = 5 per group, 200 µL/mouse). Control mice received phosphate buffer saline (PBS, Gibco) (*n* = 5 per group). Serum samples were obtained by puncturing the retro-orbital venous plexus of mice at day 31 and specific antibodies were measured by enzyme immunoassay (EIA) on antigen-coated plates (5 µg/mL, diluted in 50 mM phosphate buffer pH 7.4, 100 µL/well). Serial dilutions (from 1/50 to 1/10^6^) of individual serum from immunized mice were performed in EIA buffer (0.1 M phosphate buffer, 0.1% bovine serum albumin, 0.15 M NaCl, 0.01% sodium azide) and applied to coated plates for 18 h at +4°C. After washing (0.01 M phosphate buffer pH 7.4, 0.05% Tween 20), acetylcholinesterase (AChE)-labelled anti-IgE, IgG1 or IgG2a antibodies were applied for 3 h at room temperature as previously described [34]. Solid-phase bound AChE activity was determined by addition of 200 µL/well of Ellman's medium and absorbance measurement at 414 nm [35]. Specific IgG1 and IgG2a antibodies produced in mice receiving BLG were quantified as previously described [34]. Quantitative enzyme immunoassays for anti-Cry1Ab IgG1 and IgG2a were developed using combinations of well-characterized anti-Cry1Ab mAbs of known isotype as standard (i.e. mAbs #15, #68, #73, and #85 for IgG1 determinations and mAbs #39 and #95 for IgG2a determinations) [30], [34]. As no mAbs specific for KLH and Ara h 1 were available, semi-quantifications were performed using the same standard curves as those obtained in the same conditions for anti-BLG specific antibody determination. Concentrations of specific IgE antibodies were not quantified but results were expressed as absorbance units at 414 nm (mAU_414_nm), whatever the protein considered. Nonspecific binding (NSB) was determined using EIA buffer instead of serum. Limit of detection was determined as mean of NSB +3σ*_n_*_−1_. Mice given the different proteins were compared with mice receiving PBS, using the nonparametric Mann-Whitney test.

On day 35, all mice were sacrificed by vertebral dislocation and spleens were harvested and pooled within each treatment group. After lysis of red blood cells (180 mM NH_4_Cl, 17 mM Na_2_EDTA) and several washes, splenocytes were resuspended in RPMI-10 (RPMI supplemented with 10% fetal calf serum, 2 mM L-glutamine, 100 U penicillin, 100 µg/mL streptomycin – all from Gibco). Cells were incubated in 96-well culture plates (10^6^ cells/well) in the presence of the same protein as that administered to the mice (from 0.5 to 50 µg of protein/mL), RPMI-10 (negative control), or concanavalin A (1 µg/mL, positive control) for 60 h at 37°C and 5% CO_2_. Supernatants were then removed and stored at −80°C until further assay. IL-5 was assayed as described in [36]. IL-4 and IFNγ were assayed using commercial ELISA kits following the manufacturer's recommendations (CytoSet, Biosource International).

### 4. Analysis of the anti-maize proteins and anti-Cry 1Ab immune responses and of the metabolomic profiles after administration of MON810 maize protein extracts

#### 4.1 Preparation and characterization of whole protein extracts from MON810 and non-GM maize

Flours from grains of MON 810 maize expressing Cry1Ab (DKC6575 cultivar) and its conventional counterpart (Tietar) were used [37]. Both cultivars were grown under same conditions. Ten grams of flour were added to 20 mL of potassium phosphate buffer (50 mM, pH 7.4), and homogenized using an Ultra-Turrax grinder (Janke & Kunkel, IKA Labortechnik, Germany). After rotational shaking for 18 h at 4°C, samples were centrifuged (1000 *g*, 20 min, +4°C) and supernatants collected. Protein contents were assayed using the BCA kit from Pierce. Protein contents in the extracts were 13.7 mg/mL and 13.9 mg/mL for the samples from the GM and non-GM maize, respectively.

Qualitative and quantitative analysis of intrinsic allergens in GM and non-GM maize protein extracts was then performed using sera from self-reported maize-allergic patients 19392-CS and 20770-MH obtained from Plasmalab (WA, USA), which contained respectively 20-30 kIU/l and 20 kIU/l of corn-specific IgE as assayed using the Phadia CAP system (Pharmacia Diagnostics, Uppsala, Sweden). Electrophoresis and western blot analysis was then performed as following: 50 µL of protein extract from MON 810 or non-GM maize was mixed with 25 µL of 4X Laemmli loading buffer (1.5 M Tris pH 6.8, 10% SDS, 1% β-mercaptoethanol, 40% glycerol, 1.25% bromophenol blue) and 25 µL of deionized water and heated at 90°C for 10 min. Electrophoresis was performed by loading 12 µL of sample or SeeBlue® Plus2 Pre-stained Standard (Invitrogen) on 12% SDS-PAGE gels. Migration in Tris glycine buffer was performed at 10 mA for 120 minutes and then at 20 mA for 60 minutes, using the Mini-Protean II® cell system from BioRad (CA, USA). Proteins from one gel were stained using GelCode® Blue Reagent (Thermo Scientific, Rockford IL, USA). Proteins from other gels were transferred onto a PVDF membrane using the Mini Trans-Blot® cell from BioRad (36 V for 90 minutes) and then saturated according to the manufacturer's recommendations. The membrane was then cut into strips which were incubated for 18 h at 4°C with 1/20 diluted sera from the 2 maize-allergic patients. After washing, anti-human IgE secondary antibodies were added (goat anti-human IgE-HRP, 1/2000; STAR96P; Serotech, UK). After 1-h incubation, membranes were extensively washed and then stained with Amersham ECL plus western blotting detection reagent, following the manufacturer's recommendations. Signals were visualized by exposing a detection Kodak film to the blots and further developed with X-ray developer reagents from Kodak.

In parallel, IgE specific to GM and non-GM maize proteins were determined using enzyme allergosorbent tests (EAST) as previously described [38]. Briefly, microtiter plates were coated by passive adsorption with 5 µg/mL of GM or non-GM protein extract (50 mM phosphate buffer, pH 7.4 for 24 h at 4°C). After saturation for at least 4 h at room temperature with EIA buffer, 50 µL per well of serial dilutions of individual serum (1/5 to 1/125, in EIA buffer) was dispensed. A standard curve was determined using plates coated with an anti-human IgE (clone LE27) and standard human IgE (World Health Organization) at concentrations ranging from 10 to 0.08 IU/mL. After 24-h incubation at 4°C and a wash step, a second anti-human IgE antibody (clone BS17) labelled with AChE was used as a tracer. After extensive washings, Ellman's reagent was used as enzyme substrate and the absorbance was measured at 414 nm.

Cry1Ab concentrations were determined on serial dilutions of the protein extracts from non-GM and GM maize (from 1/100 to 1/7290) using an in-house ELISA developed within the EU-funded CoExtra Project (GM and non-GM supply chain: their co-existence and traceability) and validated using standard samples of maize grain flours containing known concentrations of MON810 maize (i.e. 0, 0.5, 1 and 2.5%, kindly provided by the Institute for Reference Materials and Measurements, EU-Joint Research Centre, Geel). Standard flour samples (250 mg) were mixed with 2 mL of extraction buffer (0.1 M sodium carbonate pH 10, 0.5 M NaCl, 0.05% Tween, 0.05% DTT and a protease inhibitor cocktail [30]). Samples were agitated for 1 h at 20°C and then centrifuged (3800 *g*, 15 min, +4°C). Supernatants were collected and Cry1Ab was assayed using a sandwich ELISA. Capture antibody (i.e. mAb #120, section 2.2) was passively immobilized on 96-well microtiter plates for 18 h at 20°C (5 µg/mL in 50 mM phosphate buffer pH 7.4, 100 µL/well). After washing and saturation, serial dilutions of standard Cry1Ab or protein extracts from non-GM and GM maize flours in EIA buffer were incubated (100 µL/well) for 2 hours at 20°C. After washing, 100 µL of anti-Cry1Ab mAb #95 (section 2.2) labelled with AChE were added to plates and incubated for 2 h at 20°C. Plates were then extensively washed and solid phase–bound AChE activity was determined by addition of 200 µL/well of Ellman's reagent [35].

#### 4.2. Analysis of the immune response in mice sensitized with GM and non-GM maize protein extracts

Three-week-old female BALB/cJ mice were fed a maize protein–free diet (Purified Diet 210, SAFE, Augy – France). Two weeks later, the mice were sensitized to protein extracts from MON810 or the non-GM counterpart. The procedures used were either i.p. administration of 100 µg of protein/mouse emulsified with incomplete Freund's adjuvant on days 1 and 15 (n = 8/group) [13] or i.g. administration of 1 mg of protein mixed with 10 µg of cholera toxin/mouse on days 1, 7, 13, 19 and 26 (n = 10/group) [12]. Ten mice received PBS via the i.g. route (PBS mice). Serum samples were obtained by puncturing the retro-orbital venous plexus of mice at day 30 and antibodies specific to maize proteins and to Cry1Ab were measured by immunoassays on antigen-coated plates, as previously described. All sera were analyzed individually at convenient dilution. After bleeding, all mice were sacrificed by vertebral dislocation and spleens were harvested and pooled within each treatment group. Cellular suspensions were prepared as previously described. Cells were incubated in 96-well culture plates in the presence of RPMI-10 or irrelevant antigen (negative control), concanavalin A (1 µg/mL, positive control), purified Cry1Ab (1, 5 or 20 µg/mL), or GM and non-GM maize protein extracts (5, 25 or 125 µg of protein/mL) for 60 h at 37°C and 5% CO_2_. Supernatants were then removed and stored at −80°C until further assay. IL-2, IL-4, IL-5, IL-10, IL-13, GM-CSF, IFNγ, TNFα and IL-17 were assayed using BioPlex technology and the mouse cytokines kit from BioRad, following the manufacturer's recommendations.

#### 4.3. Metabolomic analysis of mouse urine after i.g. administration of MON810 *vs* non-GM maize protein extracts

Metabolomics, as a holistic method used to characterize the functioning of living systems, is useful here for tentative detection of specific low-intensity metabolic effects that could be related to exposure to protein extracts from either MON810 or non-GM maize. Basically, this method uses a stringent multivariate statistical procedure to mine metabolic fingerprints obtained from a biological matrix to both discriminate groups of individuals and display significant biomarkers. Here, linear discriminant analysis (LDA) and orthogonal corrected PLS-based discriminant analysis (OPLSDA) [39]–[40] were used to discriminate the two groups of mice submitted to a time-course study of the effects of maize protein extracts. In OPLSDA, a regression model is calculated between the multivariate data and a response variable that only contains group information.

Urine samples from individual identified mice given MON 810 or non-GM maize protein extracts by the i.g. route (see above) were then collected before the first administration, and then on days 27 and 28, i.e. 24 and 48 h after the last administration. Urine samples were prepared by mixing urine (80–100 µL) with phosphate buffer (600–620 µL, pH 7.4, 0.2 M) containing 10% D_2_O as a field frequency lock and 0.05% sodium 3-trimethylsilylpropionate-2,2,3,3-*d_4_* (TMSP, internal standard) as a chemical shift reference. Buffered urine samples were then centrifuged at 13000 *g* for 10 min to remove any precipitates, and aliquots of the supernatants (600 µL) were transferred to 5 mm NMR tubes for ^1^H NMR analysis.

All ^1^H NMR spectra were recorded at 300 K on a Bruker DRX-600 Avance NMR spectrometer (Rheinstetten, Germany) operating at 600.13 MHz for ^1^H resonance frequency, using an inverse detection 5 mm ^1^H-^13^C-^15^N cryoprobe attached to a cryoplatform (the preamplifier cooling unit). The 1D “Improved Watergate” sequence was used for suppression of water resonance. Typically 128 free induction decays (FIDs) were collected into 32k data points using a spectral width of 12 ppm. The FIDs were multiplied by an exponential weighting function corresponding to a line broadening of 0.3 Hz prior to Fourier transformation. The acquired NMR spectra were phased, baseline-corrected and referenced to TMSP resonance (δ 0 ppm).

For assignment purposes, 2D correlation spectroscopy (COSY) NMR spectra were also acquired for a urine sample. Sixty-four transients per increment and 256 increments were collected into 2048 data points. The spectral width in both dimensions was 10 ppm. Before Fourier transformation, an unshifted sine-bell apodization function was applied to the free induction decays from the COSY spectra.

The NMR spectra over the range of δ 0.5–10.0 ppm were reduced by using AMIX (Bruker Analytik, Rheinstetten, Germany) to regions, each 0.01 ppm wide, and the signal intensity in each region was integrated. The region δ 4.5–6.5 ppm was removed to eliminate baseline effects of imperfect water resonance suppression. Normalization to the total sum of the spectrum was carried out on the data before multivariate statistical analysis. From the 950 initial metabolic variables extracted from spectra, the multidimensional scaling procedure was repeatedly applied to select 395 fully informative metabolic variables, to which were applied an orthogonal signal correction procedure based on a partial least squares (PLS) regression using dummy variables designed according to the “time” and “cultivar” effects. Nearly 84% of the initial metabolic information was kept after discarding on a single orthogonal component the non-modeled information from the initial dataset. One-way ANOVA considering the different groups of animals, which correspond to the combination of “cultivar” and “time” factors, was further applied at a 0.01 alpha risk and 315 variables thus selected were then submitted the Carlier algorithm [41] to select 64 uncorrelated variables, of which only the 25 most informative were used to perform multivariate analyses such as LDA and orthogonal corrected PLS-based discriminant analysis O2-PLS-DA using Splus 2000 (v2.0, Insightful Corp., Seattle, WA) including Mass and Multidim (www.lsp.ups-tlse.fr/Carlier/Logiciel.html) libraries, SAS (v8.01, SAS Institute Inc., Cary, NC) and Simca P (v 12.0, Umetrics, Umea, Sweden) software.

## Results

### 1 Analysis of the immune response induced by administration of purified proteins

The intrinsic immunogenic/allergenic potential of Cry1Ab was assessed in BALB/cJ mice by administering the purified protein by different routes without adjuvant. The humoral response (i.e. specific antibody production) and the cellular response (cytokine production by reactivated splenocytes) were analyzed and compared with the responses induced after administration of KLH, BLG and Ara h 1.

#### 1.1. Analysis of the immune response induced after i.g. administration

No specific IgE antibody production was observed after i.g. administration of either 1 or 100 µg of any of the different proteins. Low levels of specific IgG1 and/or IgG2a (<20 ng/mL) were detected in a few mice receiving 1 µg of proteins. Antibody responses were less frequent at the 100 µg than at the 1 µg dose (data not shown). At both doses, high specific secretion of IFNγ was observed in the KLH group, but no antigen-specific secretion of Th1 or Th2 cytokines by reactivated spleen cells was detected in other groups, whatever the dose (data not shown).

#### 1.2. Analysis of the immune response induced after i.p. administration

**Figure 1:**
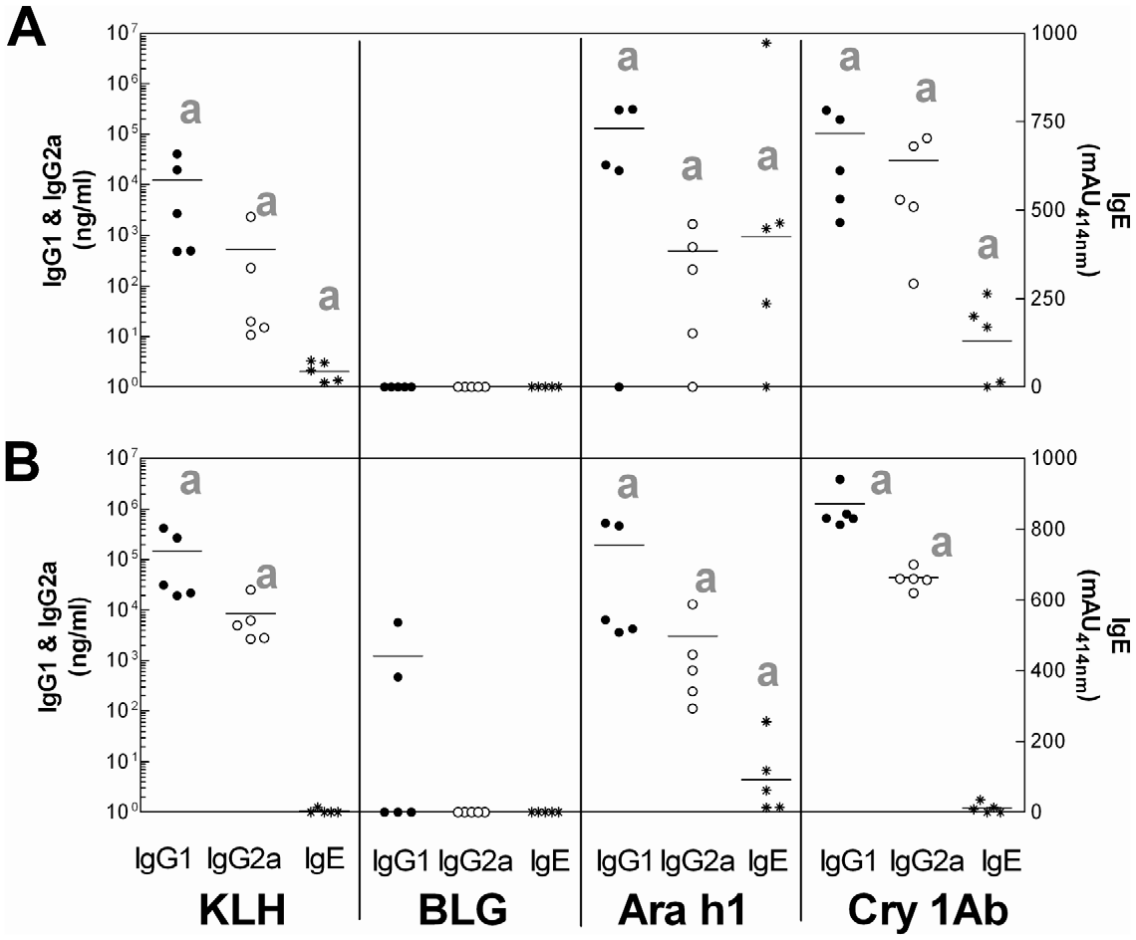
Specific IgG1, IgG2a and IgE antibodies induced after i.p. administration of purified KLH, BLG, Ara h1 or Cry1Ab. Specific IgG1 (•, left axis), IgG2a (o, left axis) and IgE (*, right axis) antibodies were assayed in sera of individual BALB/cJ mice after two i.p. administrations of 1 (**A**) or 100 µg (**B**) of each protein, without adjuvant. Bars represent the mean of 5 mice per group. Nonspecific binding (NSB) was determined using EIA buffer instead of serum. Limit of detection was determined as the mean of NSB +3σ*_n_*_−1_. a p<0.05 when comparing mice receiving protein with mice receiving PBS (not shown), nonparametric test.

I.p. administration of 1 µg of KLH induced the production of anti-KLH-specific IgG1 and IgG2a antibodies, and a weak but statistically significant specific IgE response when compared with control mice (Figure 1). After administration of 100 µg of KLH the concentrations of anti-KLH-specific IgG1 and IgG2a antibodies were approximately 10-fold higher, whereas the IgE specific response was no longer statistically significant since only one mouse out of five reacted. No significant specific antibody responses were induced after BLG administration, whatever the dose. Conversely, i.p. administration of Ara h 1 induced intense production of specific IgG1 and IgE antibodies at both doses. IgG2a antibodies were also produced at concentrations comparable to those induced after KLH administration. The specific antibody response to Cry1Ab was characterized by an intense production of anti-Cry1Ab IgG1 and IgG2a antibodies. The specific IgG1 response induced by administration of the 100 µg dosage was 20-fold higher than that induced by the 1 µg dose, while the IgG2a responses were equivalent. A low and variable but statistically significant IgE response was observed only in mice which received the 1 µg dose of Cry1Ab.

**Figure 2:**
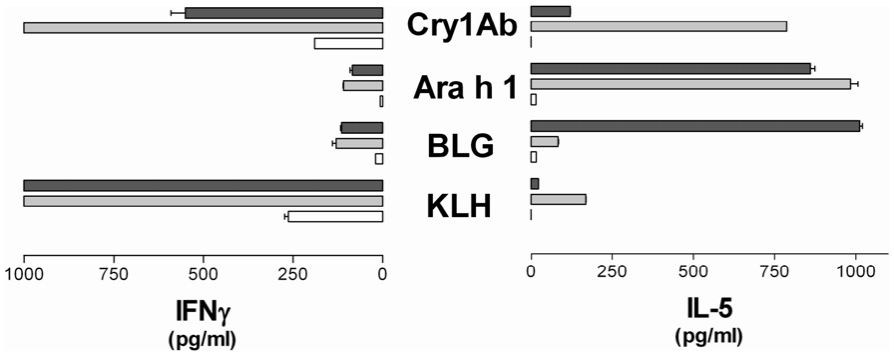
Cytokines secreted by splenocytes from mice given KLH, BLG, Ara h1 or Cry1Ab by the i.p. route. Splenocytes from BALB/cJ mice after i.p. administration of PBS (white bars), 1 µg (gray bars) or 100 µg (black bars) of KLH, BLG, Ara h1 or Cry1Ab were reactivated *ex vivo* with 50 µg of the corresponding antigen. Expected results were obtained from positive (ConA) and negative controls (medium alone) (data not shown).

After antigenic reactivation, splenocytes from mice given KLH by the i.p. route showed no or low secretion of IL-5, but intense secretion of IFNγ, whereas those from mice treated with Ara h 1 showed marked secretion of IL-5 and weak secretion of IFNγ, whatever the dose administered (Figure 2). In mice treated with BLG, IFNγ and IL-5 secretions were observed, indicating a weak specific Th1/Th2 response at the 1 µg dosage with an increased polarization toward a Th2 response at the 100 µg dose, although no specific antibody production was detected in the sera of these mice. Spleen cells from mice given 1 µg Cry1Ab showed strong IL-5 and IFNγ secretions, but both IL5 and IFNγ secretions were decreased after administration of the 100 µg dose, IL5 secretion then being similar to that induced by the 1 µg dose of KLH.

### 2. Effects of administration of MON810 maize to mice

#### 2.1 Characterization of whole-protein extracts

**Figure 3:**
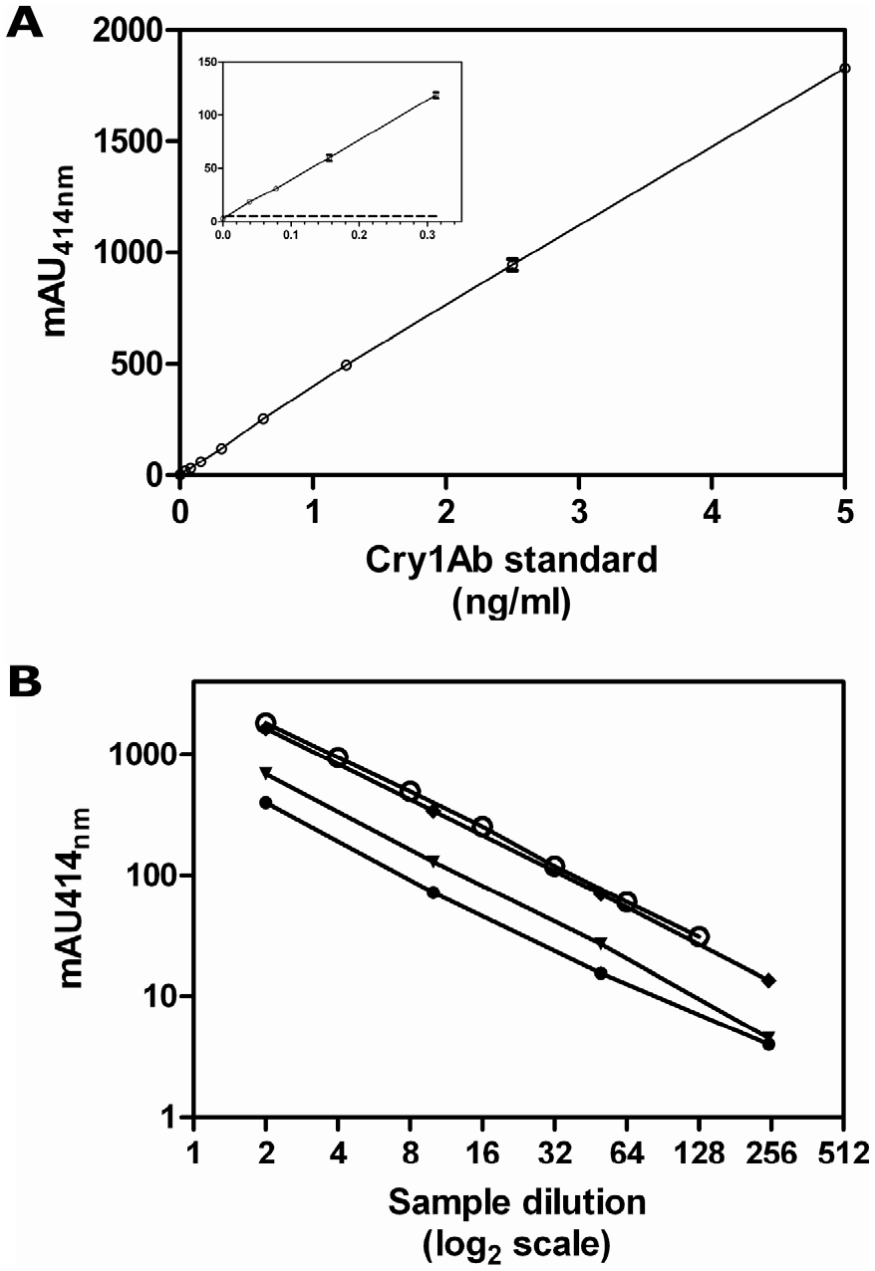
Sandwich immunoassay of Cry1Ab. **A.** Standard curve obtained with purified Cry1Ab protoxin in sandwich immunoassay using mAb#120 passively adsorbed on microplates as capture antibody and AChE-labeled mAb#95 as tracer. Mean +/− SD is represented. Each point is the result of duplicate measurements, except NSB (EIA buffer alone, n = 8). The limit of detection, defined as the lowest concentration of standard Cry1Ab inducing a signal statistically significantly higher than NSB (i.e. mean+3σ*_n_*_−1_) is shown in the insert as a black line. **B.** Extracts from reference maize powders containing MON810 maize mass fraction level of 0.5% (●), 1% (▼) or 2.5% (◆) (2- to 250-fold dilutions) or standard Cry1Ab (10 ng/mL, 2- to 128-fold dilutions, ○) were assayed using this sandwich immunoassay. All dilution points were assayed in duplicate. Results are expressed as mean of absorbance values (mAU at 414 nm) +/− SD. No signal was detected for non-GM reference maize (not shown).

Before administration to mice, both maize protein extracts were characterized in terms of their Cry1Ab contents and intrinsic allergen contents and profiles. The standard curve obtained with purified Cry1Ab in the sandwich immunoassay we have developed is shown in Figure 3A. The calculated limit of quantification (mean NSB+10σ*_n_*_−1_) is 10 pg/mL of Cry1Ab, and convenient precision of this ELISA was also obtained, as assessed by determining the intra- and inter-assay coefficients of variation (CV<10%, not shown). Dilution curves obtained with the reference materials containing known concentrations of either MON810 or standard purified Cry1Ab are shown in Figure 3B. No signal was detected with the non-GM reference material (not shown). Curves obtained are parallel, which demonstrates the efficiency and specificity of the assay. Cry1Ab concentrations measured were 26.96, 48.7, and 126.4 ng/mg of maize powder in the reference samples containing MON810 maize at concentration levels of 0.5, 1 and 2.5%, respectively, demonstrating the accuracy of the assay. Using this assay, extracts administered to mice were characterized for their Cry1Ab contents. No Cry1Ab was detected in the extract from the non-GM maize (Tietar). MON 810 extract (DKC6575 cultivar) contained 0.18 µg/mL Cry1Ab, corresponding to 0.0013% of the protein content.

**Figure 4:**
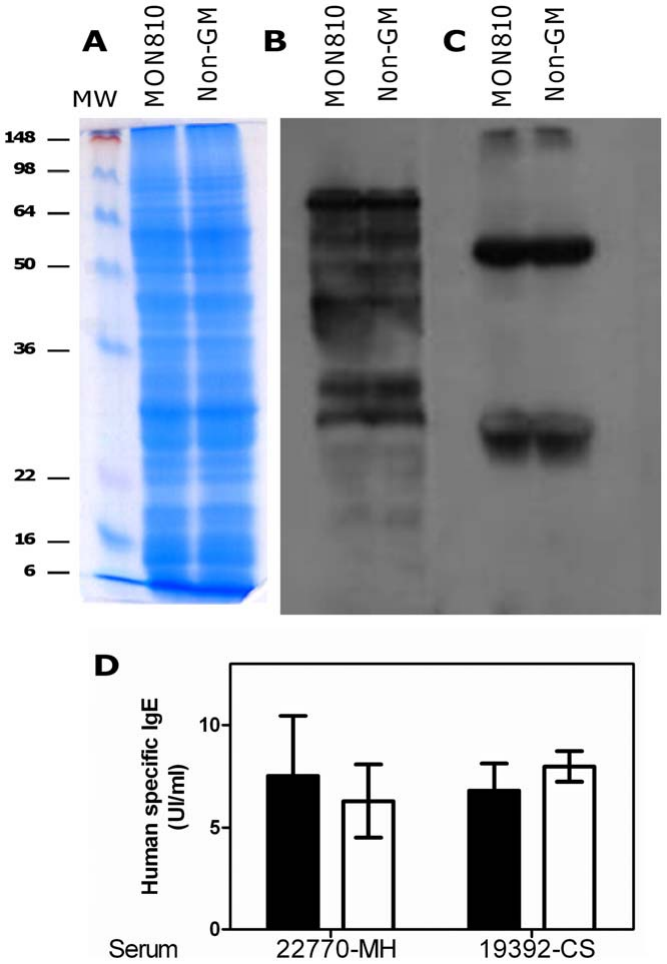
A–C: Electrophoretic pattern and IgE-binding analysis of protein extracts from MON810 and non-GM maize: Protein extracts from MON 810 and its conventional counterpart were separated on 12% acrylamide gel and proteins were stained (**A**). After transfer to PVDF membrane, IgE binding proteins (i.e. allergens) were revealed by western blotting with the sera of 2 maize-allergic patients, i.e. #20770-MH (**B**) and #19392-CS (**C**). Specific IgE concentrations (**D**) in the same sera were determined on GM (open bar) and non-GM (black bar) protein extract coated plates. Bars represent mean+/−SEM obtained for 3 different dilutions of each serum, each assayed in duplicate.

The allergen repertoires of the GM and non-GM maize were then compared by electrophoresis (Figure 4A) and immunoblotting using 2 sera from maize-allergic patients (Figure 4B–C). The 2 sera displayed different patterns of recognition by IgE antibodies, but for each serum no differences were observed between GM and non-GM maize. Moreover, concentrations of human specific IgE assayed on plates coated with GM or non-GM maize protein extract were comparable (Figure 4D). These results thus indicate that the genetic modification did not result in unintended qualitative and/or quantitative modification of expression of intrinsic allergens.

#### 2.2. Specific anti-maize and anti-Cry1Ab immune response after administration of MON810 maize

**Figure 5:**
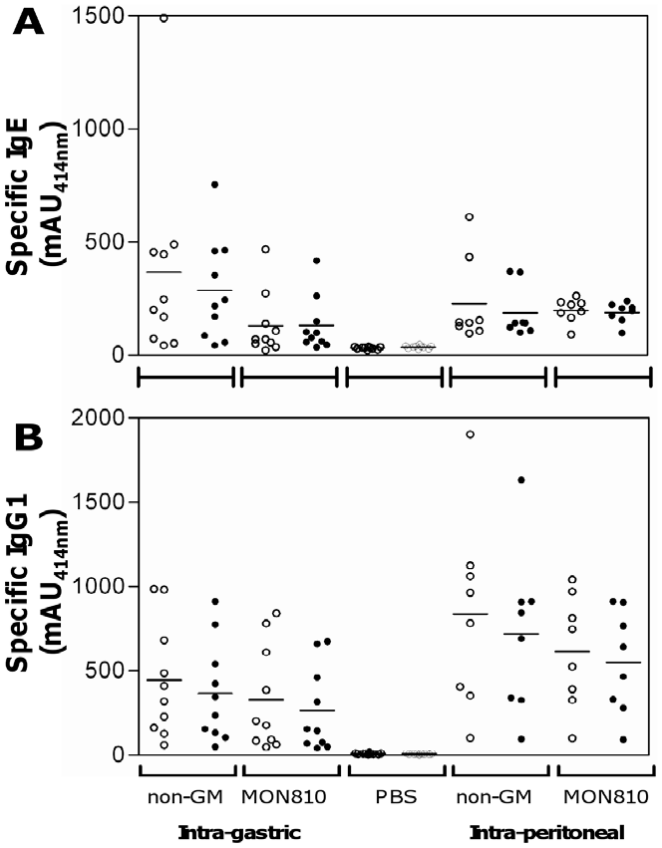
Specific IgE and IgG1 antibodies induced after i.g. or i.p. administration of GM vs non-GM maize extracts. Mice were given protein extracts from MON 810 or non-GM comparator via the i.p. route (100 µg of protein/mice emulsified with incomplete Freund's adjuvant) on days 1 and 15 (n = 8/group) or via the i.g. route (1 mg of protein mixed with 10 µg of cholera toxin) on days 1, 7, 13, 19 and 25 (n = 10/group). Ten mice received PBS via the i.g. route (PBS mice). Specific IgE (**A**) and IgG1 (**B**) antibodies were assayed on microtiter plates coated with non-GM (open circles) or MON810 (black symbols) maize protein extracts. For each group, data of individual serum samples collected on day 30 and the corresponding median values are expressed as absorbance values (mAU at 414 nm). Sample dilutions were 1/40 for assays of IgE antibodies, and 1/4000 (i.g. treated groups) or 1/200000 (i.p. treated groups) for assays of IgG1 antibodies.

Characterized maize protein extracts were then administered to mice either with cholera toxin (i.g. route) or IFA (i.p. route), two protocols that efficiently sensitize BALB/c mice to whole foods [12], [13]. The immune responses induced by GM *vs* non-GM maize were then compared within each protocol of sensitization. In each case, there was significant production of IgE (Figure 5A) and IgG1 (Figure 5B) antibodies specific to maize proteins, compared with PBS mice (p<0.05, Kruskal-Wallis test and Dunn's multiple comparison test). This demonstrated that the mice were efficiently sensitized to maize proteins in our experimental conditions. For each route of administration, no differences in IgE and IgG1 antibody responses to maize proteins were observed between mice treated with the GM or non-GM maize. No difference in determination was observed whatever the ELISA procedure used, i.e. whatever the extract immobilized on the microtiter plate (Figure 5). In the same way, no significant difference in Th1, Th2 or Th17 related–cytokine secretion was seen after GM and non-GM maize protein extract restimulation of splenocytes from the treated animals, whatever the route and extract used for immunization (data not shown). No specific anti-Cry1Ab antibody was detected in serum from mice given the GM maize extract, after either i.g. or i.p. sensitization, and no cytokine production was detected after Cry1Ab reactivation of splenocytes from the corresponding mice.

#### 2.3 Metabolomic analysis of urine from mice given maize protein extracts by the i.g. route

**Figure 6:**
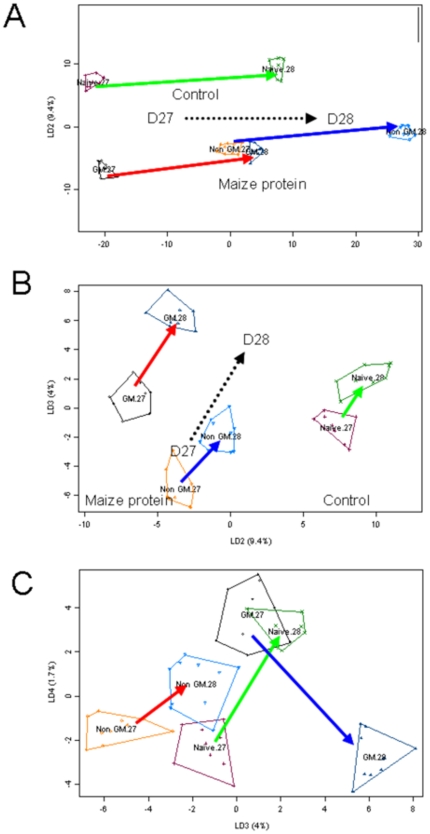
Linear discriminant analysis performed on OSC-PLS–corrected data corresponding to the late urine collections performed on days 27 and 28. The four LD are highly significant (p<0.0001) and the 3 respective factorial maps are displayed here: 1×2 (**A**), 2×3 (**B**) and 3×4 (**C**). The cultivar names are displayed on the factorial maps with the .2 and .3 suffixes designating the urine collection performed on days 27 and 28, respectively.

As a complement to the targeted analysis of the immune response, we used a non-targeted approach using metabolomics to detect any unintended and unexpected effects provoked by i.g. exposure to GM maize protein extracts. ^1^H NMR fingerprinting of urine collected from individual mice before i.g. administration and either 24 or 48 h after the last administration (i.e. on Days 27 and 28) gave a time-dependent progression of metabolic signatures which accounted for 86% of the between-group variance (1^st^ discriminant axis) and a difference between control and maize protein administration (6.3% of the between-group variance, 2^nd^ discriminant axis), with no difference resulting from the administration of the GM *vs* the non-GM (not shown). When this first data set was restricted to the study of the final experimental situation after the 5^th^ i.g. administration on Day 26, it was possible to detect more subtle but significant metabolic variations. With such a data set, for which a prior orthogonal signal correction based on “time” and “cultivar” factors is made, keeping 49.4% of the initial information, it is possible again to detect a significant change in the metabolic signature between the 27^th^ and 28^th^ days, which represents 84.2% of the between-group variance and is explained by the 1^st^ linear discriminant axis (LD1, p<0.0001) (Figure 6A). Interestingly, the LD2, which accounts for 9.4% of the between-group variance, discriminates between control animals and maize protein–treated ones (p<0.0001). As for LD1, the 3^rd^ LD (LD3) with 4% of the variance is well explained by the day-to-day variation of the mouse metabolome (p<0.0001). This likely explains the clear opposition on the factorial projection between the group of animals treated with non-GM maize proteins, which were analyzed on the 27^th^ day, and the group of animals treated with MON 810 proteins, which were analyzed on the 28^th^ day (Figure 6B). It is only on the 4^th^ LD explaining 1.7% of the between-group variance that metabolomic variation depending only on the cultivar factor is seen (p<0.0001, Figure 6C). On the factorial plan 3×4, the trajectory from D27 to D28 for mice treated with non-GM maize proteins is parallel to that of control animals, but orthogonal to the trajectory of mice treated with MON 810 proteins. The same LD axes were found with an O2-PLS discriminant analysis, which is appropriate to the data treatment of such a low rank variance-covariance matrix (not shown).

Metabolic biomarkers were identified from this discriminant analysis. Following the last administration on day 26, a relative general decrease in urinary excretion of myo-inositol (ρ = −0.97), hippurate (ρ = −0.87) and taurine (ρ = −0.78) was seen, with a parallel increase in creatine (ρ = 0.98) (Table 1, axis 1). The second discriminant axis explains the differentiation between maize-treated mice and control mice, with a relative increase in urinary excretion of threonine (ρ = −0.96) and phosphocholine (ρ = −0.85) and a relative decrease in taurine excretion in maize protein–treated mice (ρ = 0.91). The biomarker-based interpretation of the last two LD is more complex. The difference between the mice treated with the non-GM maize proteins and those treated with the MON 810 proteins is well explained considering the LD3 by a lower excretion of both phenylalanine (ρ = −0.83) and hippurate (ρ = −0.53) and a higher excretion of tryptophan (ρ = 0.53) in the latter mice. In parallel, the difference between MON 810–treated mice analyzed on day 27 and the same mice analyzed on day 28 is well explained by a relative increase in excretion of some dicarboxylic acids (ρ = −0.88), taurine (ρ = −0.48) and triethylamine (ρ = −0.37) in the latter group (Table 1, axes 3 and 4). All these observations cannot be simply and directly related to immune or inflammation biomarkers.

**Table 1:** Metabolic biomarkers identified from discriminant analysis.

Chemical shift (ppm)	Assignment	Axis 1		Axis 2		Axis 3		Axis 4	
		ρ	rank	ρ	rank	ρ	rank	ρ	rank
3.93	creatine	0.98	1	0.19		−0.01		0.05	
4.09	myo-inositol	−0.97	2	−0.20		0.08		−0.09	
3.87	glycogen (?)	−0.91	3	0.14		−0.22		0.28	
7.55	hippurate	−0.87	4	0.25		−0.25		−0.34	
3.61	myo-inositol/glycogen	−0.85	5	−0.12		−0.27		0.37	
3.20	choline	−0.80	6	0.18		−0.48	7	−0.31	
3.40	taurine	−0.78	7	0.58	11	0.05		−0.20	
4.25	threonine	0.004		−0.96	1	−0.01		−0.25	
3.29	taurine	−0.34		0.91	2	0.11		−0.20	
4.18	phosphocholine	−0.001		−0.85	3	0.50	6	0.10	
3.30	taurine	−0.38		0.76	4	0.15		−0.48	4
4.16	lactate	0.33		−0.76	5	0.43		0.29	
7.27	phenylalanine	0.06		−0.21		−0.83	1	0.48	5
3.68	?	−0.004		−0.52	13	−0.60	2	0.51	2
7.69	tryptophan	0.77	8	−0.28		0.54	3	0.22	
7.85	hippurate	−0.59	9	−0.52	12	−0.53	4	0.29	
4.17	lactate	−0.44		−0.72	6	0.53	5	0.04	
0.85	dicarboxylic acids	−0.22		0.38		−0.15		−0.88	1
1.89	arginine/lysine	−0.35		0.69	7	0.22		−0.50	3
2.06	N-acetylglycoproteins	−0.34		−0.68	9	0.32		0.38	6
2.88	trimethylamine	−0.45	11	0.69	8	−0.09		−0.37	7
1.59	dicarboxylic acids	0.53	10	−0.58	10	0.43		0.34	8

Assignment of significant variables explaining the 4 first components displayed by a linear discriminant analysis performed on OSC-PLS–corrected data corresponding to the late urine collections performed on days 27 and 28.

## Discussion

To analyze the intrinsic immunogenicity and potential impact of exposure to Cry1Ab protein on the immune response of mice, we first performed i.g. or i.p. administration of the purified protein to BALB/c mice without adjuvant. We compared the antibody response and cytokine secretion profile of mice receiving Cry1Ab with those induced by administration of KLH (a highly immunogenic protein not known to be an allergen), BLG (a moderate allergen) or Ara h1 (a strong allergen) [14]. We did not detect significant amounts of specific antibodies after i.g. administration of any of the proteins. Notably, no anti-Cry1Ab IgG1 or IgG2a antibodies were detected after i.g. administration of 100 µg Cry1Ab, whereas such responses were induced in mice receiving the same doses of Cry1Ac by this route according to [20]. This is surprising considering the 86% amino acid sequence homology between Cry1Ab and Cry1Ac proteins and is likely due to the presence of endotoxin contaminating the bacterial preparation of recombinant Cry1Ac used in [20]. Conversely, i.p. administration of the different proteins generally resulted in the induction of an immune response. In the present study, 100 µg and 1 µg of KLH or Cry1Ab induced only a Th1 or a mixed Th1/Th2 response, respectively. The administration of BLG induced only a weak cellular Th2 response at the dose of 100 µg, whereas that of Ara h1 induced a strong Th2 response whatever the dose administered. A clear Th2 response was then observed for the strong allergen Ara h 1, whatever the dose, and to a lesser extent for BLG. The anti-Cry 1Ab immune response could not be distinguished from that induced by KLH, which is considered as a nonallergenic protein. Administration of low doses of proteins has previously been reported to favor the induction of IgE in BALB/c mice independently of the intrinsic allergenicity of the protein [42]–[43]. It appears that high levels of antigen result in selective stimulation of Th1 cells, which produce IFNγ, and diminished activation of IL-4-producing Th2 cells [44]. The observed mixed Th1/Th2 response at low doses of Cry1Ab and KLH should then reflect the protein immunogenicity in a Th2-biased strain of mice rather than allergenicity. Conversely, the clear Th2 response observed after administration of high doses of Ara h 1 or BLG reflects their intrinsic allergenicity. Our results in the BALB/c mouse thus show that i.g. administration of purified Cry1Ab had no impact on the immune response and confirmed previous studies reporting its immunogenicity after i.p. administration, without evidencing allergenicity.

Cry1Ab is expressed at very low levels in MON810 maize, i.e. ca. 0.0013% of the protein content in the present study. Therefore we also investigated the immune response to Cry1Ab administered within MON810 grains. We first tried to detect any possible unintended effect of the genetic modification on the allergenicity of the whole crop that might result in alteration of the quantitative and qualitative expression patterns of endogenous maize allergens in MON810 *vs* its conventional counterpart. No differences in the allergen repertoire were detected by IgE-immunoblot studies using sera from 2 maize-allergic patients. This confirms the results of the gene expression profiling studies performed on leaves of the same GM and non-GM lines [37]. The immune response was then analyzed in mice experimentally sensitized by i.p. or i.g. administration of whole protein extracts from GM or non-GM maize in the presence of appropriate adjuvants. All mice were sensitized to maize proteins as demonstrated by the production of specific IgE and IgG1 antibodies against maize proteins and Th2 cytokine secretions by reactivated spleen cells, but no differences in anti-maize protein specific IgE and IgG1 titers were observed after i.p. or i.g. administration of whole protein extracts from either the GM or the non-GM maize. In addition, no anti-Cry1Ab humoral and cellular immune responses were detected in mice that received the protein extract from MON810 maize.

Metabolomics easily enables quantitative and dynamic study of the perturbation of living systems during or after exposure to compounds that may have biological impacts [45]. In the present study, metabolomics was used as a non-targeted approach to detect any unintended and unexpected effects of the GM maize protein extract. It was considered complementary to the targeted analysis of the immune response, to allow comprehensive assessment of the impact of exposure to GM maize in mice, although both approaches might not be directly related. Clearly, no early biomarkers of immunological or inflammatory processes were observed. Effects of the repeated intra-gastric administrations of maize proteins accounted for less than 10% of the complete metabolic variance analyzed in this experiment, the main factor accounting for the change in the metabolic fingerprint being the age of the animals. A parallel progression of the urinary metabolic pattern of mice treated with the 2 maize protein extracts is shown on the 2^nd^ and 3^rd^ discriminant axes. It is only with the 4^th^ axis that a “cultivar” effect is detectable, which represented 1.7% of the metabolic information. This figure was calculated using an OSC-PLS–based data correction, and hence represents less than 1% of the initial metabolic information. This so-called “cultivar” effect includes both the influence of the natural genetic background of the 2 maize lines (i.e. MON810 and the conventional counterpart) and the possible impact of the genetic modification itself. The latter effect, if any, would thus account for less than 1% of the metabolic variance.

In conclusion, using the BALB/c mouse model in particular experimental conditions of exposure, we have demonstrated that Cry1Ab is immunogenic and may induce a mixed Th1/Th2 immune response when it is administered as a purified protein by the i.p. route. The same response was observed when considering KLH and no allergenic potency could then be evidenced for Cry1Ab. This effect was not observed after administration of a protein extract from MON810 which resulted in an immune response against maize proteins but not against Cry 1Ab. Metabolomic studies on urine from treated mice (i.g. route) showed a low “cultivar” effect, which represented 1.7% of the OSC-corrected metabolic information, and in which the genetic modification would account for less than 1%. More GM and non-GM cultivars should be included in targeted studies to achieve a more detailed analysis and interpret a possible biological significance of this effect.
